# Prescription Drugs Associated with Reports of Violence Towards Others

**DOI:** 10.1371/journal.pone.0015337

**Published:** 2010-12-15

**Authors:** Thomas J. Moore, Joseph Glenmullen, Curt D. Furberg

**Affiliations:** 1 Institute for Safe Medication Practices, Alexandria, Virginia, United States of America; 2 Department of Psychiatry-Cambridge Hospital, Harvard Medical School, Cambridge, Massachusetts, United States of America; 3 Division of Public Health Sciences, Wake Forest University School of Medicine, Winston-Salem, North Carolina, United States of America; Yale University School of Medicine, United States of America

## Abstract

**Context:**

Violence towards others is a seldom-studied adverse drug event and an atypical one because the risk of injury extends to others.

**Objective:**

To identify the primary suspects in adverse drug event reports describing thoughts or acts of violence towards others, and assess the strength of the association.

**Methodology:**

From the Food and Drug Administration (FDA) Adverse Event Reporting System (AERS) data, we extracted all serious adverse event reports for drugs with 200 or more cases received from 2004 through September 2009. We identified any case report indicating homicide, homicidal ideation, physical assault, physical abuse or violence related symptoms.

**Main Outcome Measures:**

Disproportionality in reporting was defined as a) 5 or more violence case reports, b) at least twice the number of reports expected given the volume of overall reports for that drug, c) a χ2 statistic indicating the violence cases were unlikely to have occurred by chance (p<0.01).

**Results:**

We identified 1527 cases of violence disproportionally reported for 31 drugs. Primary suspect drugs included varenicline (an aid to smoking cessation), 11 antidepressants, 6 sedative/hypnotics and 3 drugs for attention deficit hyperactivity disorder. The evidence of an association was weaker and mixed for antipsychotic drugs and absent for all but 1 anticonvulsant/mood stabilizer. Two or fewer violence cases were reported for 435/484 (84.7%) of all evaluable drugs suggesting that an association with this adverse event is unlikely for these drugs.

**Conclusions:**

Acts of violence towards others are a genuine and serious adverse drug event associated with a relatively small group of drugs. Varenicline, which increases the availability of dopamine, and antidepressants with serotonergic effects were the most strongly and consistently implicated drugs. Prospective studies to evaluate systematically this side effect are needed to establish the incidence, confirm differences among drugs and identify additional common features.

## Introduction

Violent thoughts and acts towards others area common occurrence in our society but rarely studied as an adverse drug event. Increased risk of suicidal behaviors—but not violence— associated with antidepressants has been examined through meta-analysis of clinical trials for approval by the U.S. Food and Drug Administration. [1], [2]

Despite limited clinical study, numerous drugs contain FDA-required warnings to doctors or patients about the possibility of aggressive or violent acts. Among the drugs with warnings about aggressive behaviors are varenicline, zolpidem, montelukast, and all antidepressant drugs. [3]–[6] The mandatory patient Medication Guide for varenicline, the antidepressants and quetiapine warn patients to contact a healthcare provider immediately if they start “acting aggressive, being angry or violent.” [3], [7]–[9]

In this study we summarize and evaluate the evidence about reported acts of violence associated with therapeutic drugs among all serious adverse drug events reported to the FDA from 2004 through the third quarter of 2009.

## Methods

The cases for this study were selected from the Institute for Safe Medication Practices (ISMP) QuarterWatch database of all adverse drug events reported to the FDA since 1968. [10] The FDA publishes for research use computer extracts of all adverse drug event reports that it receives, [11] and all such cases are included in the QuarterWatch database. While best known to medical professionals as “MedWatch Reports,” the FDA's adverse event database also includes serious foreign cases from international drug companies who market the drugs in the United States. We limited this study to cases with serious outcome as defined by the FDA, and which includes death, disability, hospitalization, a life threatening event, an event that required medical intervention to prevent harm, or other medically serious conditions. The latest version of all cases with an initial report date from 2004 through the third quarter of 2009 was included. To qualify for inclusion in this study as an evaluable drug, it had to have wide enough clinical use and sufficient post marketing surveillance to have generated 200 or more case reports for any serious adverse event in the study period. Drug names were standardized from the QuarterWatch dictionary, which is in turn based on standardized ingredient names in the National Library of Medicine RxNorm database. [12]

### Identification of Violence Reports

In the published computer extracts, the adverse event narrative description is replaced by one or more standardized medical terms selected from Version 11.1 of the Medical Dictionary for Regulatory Affairs (MedDRA). [13] We defined a violence event as any case report containing one or more of the following MedDRA terms: Homicide, Physical assault, Physical abuse, Homicidal ideation or Violence-related symptom. If a case report contained more than one of these terms, it was assigned to the most severe event term in the order listed above. In selecting terms from the MedDRA dictionary, we sacrificed some degree of sensitivity in case identification to achieve greater specificity. Thus, from the High Level Term group “Criminal Activity” we selected Homicide and Physical assault but omitted more ambiguous descriptors such as “Crime” or “Spousal Abuse.” Similarly from the High Level Term group Behavioral and Social Disturbances we selected Violence-related Symptom and Homicidal ideation but omitted the more general terms Aggression, Belligerence and Hostility. To assure an accurate count of widely used drugs for which no violence cases were reported we included all evaluable drugs in our data set.

### Proportional Reporting Ratio

The null hypothesis for this study assumed that a violence case could be attributed to a drug by pure chance, and that drugs with a greater total number of adverse event reports might be exposed to greater risk of accruing a violence case. A drug might have a larger total number of adverse events reported for several reasons unrelated to safety, including more widespread clinical use, a higher reporting rate, the patient population treated, and more extensive international sales by the pharmaceutical sponsor.

To assess the null hypothesis we calculated the proportional reporting ratio (PRR) for each evaluable drug using the method of Evans. [14] With this method we compare the proportion of violence cases for each drug (drug violence events/all drug events) to the proportion of all other violence events for all other evaluable drugs (all other violence events/all other drugs events). For example, violence cases accounted for 35/3689 (0.95%) of all cases for the drug bupropion. For all the other drugs, violence cases accounted for 1902/776480 (0.2%) PRR = 3.9. In other words, the number of violence cases was 3.9 times greater for bupropion than for all other drugs combined after adjusting for the volume of reports. Using the χ^2^ test for independence, we assessed the strength of the relationship, and the probability that that the PRR could have occurred by chance. In the bupropion example, χ^2^ = 70.6 df = 1 p<0.01. To allow for the fact that some drugs might have as few as 5 index cases we applied the Yates correction to the χ^2^ statistic.

### Study Criteria

To be identified as disproportionally associated with violence cases, an evaluable drug had to have a PRR≥2, a χ^2^≥4.0, and 5 or more violence cases attributed. These are the Evans criteria except that our minimum threshold was 5 cases rather than the 3 cases specified in Evans. As a practical matter a χ^2^≥4 with 1 degree of freedom produces a sample estimate with p<0.01 but p values are provided to assess borderline cases.

The data for this study were maintained in an open source MySQL database (Oracle, www.mysql.com) and analyzed with open source software from the R-Project for Statistical Computing (www.r-project.org), version 2-11-0.

## Results

In the 69-month reporting period we identified 484 evaluable drugs that accounted for 780,169 serious adverse event reports of all kinds. This total included 1,937 (0.25%) cases meeting the violence criteria. The violence cases included 387 reports of homicide, 404 physical assaults, 27 cases indicating physical abuse, 896 homicidal ideation reports, and 223 cases described as violence-related symptoms. The patients were 41% female and 59% male with a mean age of 36 years (SD = 17.9) Consumers reported 651/1937 (38%) of the cases, foreign and domestic health professionals were the source for 967/1937 (49.9%) and the remainder were missing (217), from lawyers (67) or clinical studies (34).

### Drugs Identified

Among 484 evaluable drugs, 31 drugs met the study criteria for a disproportionate association with violence, and accounted for 1527/1937 (79%) of the violence cases. The drugs are listed in Table 1. They include varenicline (a smoking cessation aid), 11 antidepressant drugs, 3 drugs for attention deficit hyperactivity disorder, and 5 hypnotic/sedatives. No violence cases were reported for 324/484 (66.9%) of all evaluable drugs, and 1 or 2 cases were reported for an additional 86/484 (17.8%). Thus, for 84.7% of all evaluable drugs in widespread clinical use, an association with violence appeared highly unlikely.

**Table 1:** Drugs associated with violence adverse drug events.

Drug Name	PRR*	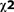 (df = 1)	P Value	Violence Cases
VARENICLINE	18.0	5172.0	p<0.01	408
FLUOXETINE	10.9	615.7	p<0.01	72
PAROXETINE	10.3	1353.2	p<0.01	177
AMPHETAMINES	9.6	227.7	p<0.01	31
MEFLOQUINE	9.5	67.2	p<0.01	10
ATOMOXETINE	9.0	339.5	p<0.01	50
TRIAZOLAM	8.7	40.3	p<0.01	7
FLUVOXAMINE	8.4	25.8	p<0.01	5
VENLAFAXINE	8.3	517.2	p<0.01	85
DESVENLAFAXINE	7.9	41.4	p<0.01	8
MONTELUKAST	7.0	260.8	p<0.01	53
SERTRALINE	6.7	297.4	p<0.01	64
ZOLPIDEM	6.7	222.1	p<0.01	48
ESCITALOPRAM	5.0	94.0	p<0.01	31
SODIUM OXYBATE	4.9	15.0	p<0.01	6
CITALOPRAM	4.3	83.1	p<0.01	34
ARIPIPRAZOLE	4.2	51.6	p<0.01	23
OXYCODONE	4.1	104.4	p<0.01	46
BUPROPION	3.9	70.6	p<0.01	35
ZIPRASIDONE	3.8	36.7	p<0.01	19
METHYLPHENIDATE	3.6	48.5	p<0.01	27
MIRTAZAPINE	3.4	22.4	p<0.01	15
GABAPENTIN	3.3	53.8	p<0.01	35
LEVETIRACETAM	3.3	30.3	p<0.01	21
DIAZEPAM	3.1	13.7	p<0.01	11
ALPRAZOLAM	3.0	17.5	p<0.01	15
DULOXETINE	2.8	49.7	p<0.01	45
CLONAZEPAM	2.8	9.5	p<0.01	10
INTERFERON ALFA	2.7	56.7	p<0.01	54
RISPERIDONE	2.2	16.6	p<0.01	29
QUETIAPINE	2.0	25.8	p<0.01	53

*Proportional Reporting Ratio.

### Smoking Cessation

Table 2 shows the results for three drugs available as an aid to smoking cessation programs: varenicline, bupropion and nicotine replacement products. Bupropion is indicated for both depression and as an aid to smoking cessation, so those results are not limited to the smoking cessation population. Varenicline has the largest number of reported violence cases, the highest proportion of violence cases (PRR = 18.0) and the highest χ2 statistic (χ2 = 5172 df = 1 p<0.01) of any of the 484 evaluable drugs. Comparing reports of violence in the patient population seeking to discontinue smoking provides adjustment for the possibility that the side effects might be caused by stopping smoking rather than the drug.

**Table 2:** Drugs prescribed for smoking cessation.

Drug Name	PRR*	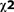	P Value	Violence Cases	All Cases
VARENICLINE	18.0	5172	p<0.01	408	11393
BUPROPION**	3.9	70.6	p<0.01	35	3689
NICOTINE	1.9	3.6	p = 0.059	11	2383

*Proportional Reporting Ratio.

**All indications of this antidepressant drug.

### Psychoactive Drugs

Since psychoactive drugs accounted for most of the drugs identified we further analyzed them by class, including other drugs in the class that did not meet the criteria for an association. The results by drug class are shown in Table 3. These data indicate marked differences between drug classes. All the antidepressants were associated with violence cases, except for two tricyclics, trazodone and amitriptyline, which had a similarly elevated PRR that was statistically significant, but fewer than 5 required cases to qualify as suspect under our criteria. The PRR for all antidepressants combined was 8.4, higher than for any other class of psychoactive drugs. At the other extreme, mood stabilizers/anticonvulsants were not implicated with the exception of levetiracetam and overall the group did not indicate an elevated risk. Results for the antipsychotics were mixed, ranging from a PRR = 4.2 for aripiprazole to a low of PRR = 0.6 for clozapine indicating fewer than expected reports (χ 2 = 1.6 p = 0.203). Grouped together the antipsychotics were borderline with a PRR of 1.9, slightly less than the 2.0 required, and χ2 = 68.5 p<0.01. The results for hypnotics/sedatives were also mixed with strong signals for zolpidem and triazolam, and little or no evidence for lorazepam and midazolam. Among the opioids, only oxycodone showed an association.

**Table 3:** Psychoactive drugs by drug class and association with violence.*

Drug Class/Drug	PRR	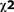	P Value	Violence	All Cause
**Antidepressants**	8.4	2956.3	p<0.01	578	37632
** FLUOXETINE**	**10.9**	**615.7**	**p<0.01**	**72**	**2752**
** PAROXETINE**	**10.3**	**1353.2**	**p<0.01**	**177**	**7508**
** FLUVOXAMINE**	**8.4**	**25.8**	**p<0.01**	**5**	**239**
** VENLAFAXINE**	**8.3**	**517.2**	**p<0.01**	**85**	**4283**
** DESVENLAFAXINE**	**7.9**	**41.4**	**p<0.01**	**8**	**410**
** SERTRALINE**	**6.7**	**297.4**	**p<0.01**	**64**	**3938**
** ESCITALOPRAM**	**5.0**	**94**	**p<0.01**	**31**	**2529**
** CITALOPRAM**	**4.3**	**83.1**	**p<0.01**	**34**	**3192**
AMITRIPTYLINE	4.2	4.5	p = 0.033	3	286
** BUPROPION**	**3.9**	**70.6**	**p<0.01**	**35**	**3689**
TRAZODONE	3.5	4.9	p = 0.026	4	457
** MIRTAZAPINE**	**3.4**	**22.4**	**p<0.01**	**15**	**1810**
** DULOXETINE**	**2.8**	**49.7**	**p<0.01**	**45**	**6539**
**Attention deficit hyperactivity**	6.9	509.4	p<0.01	108	6627
** AMPHETAMINES**	**9.6**	**227.7**	**p<0.01**	**31**	**1317**
** ATOMOXETINE**	**9.0**	**339.5**	**p<0.01**	**50**	**2290**
** METHYLPHENIDATE**	**3.6**	**48.5**	**p<0.01**	**27**	**3020**
**Mood Stabilizer/Anticonvulsant**	1.1	0.3	p = 0.585	60	22346
** LEVETIRACETAM**	**3.3**	**30.3**	**p<0.01**	**21**	**2619**
OXCARBAZEPINE	1.4	0.2	p = 0.673	5	1487
TOPIRAMATE	1.3	0.4	p = 0.505	9	2727
LAMOTRIGINE	0.8	0.2	p = 0.658	11	5275
VALPROIC ACID	0.8	0.1	p = 0.735	8	3843
PHENYTOIN	0.4	1.7	p = 0.19	3	2795
CARBAMAZEPINE	0.3	3.3	p = 0.068	3	3600
**Antipsychotic**	1.9	68.5	p<0.01	167	36225
** ARIPIPRAZOLE**	**4.2**	**51.6**	**p<0.01**	**23**	**2248**
** ZIPRASIDONE**	**3.8**	**36.7**	**p<0.01**	**19**	**2012**
** RISPERIDONE**	**2.2**	**16.6**	**p<0.01**	**29**	**5464**
** QUETIAPINE**	**2.0**	**25.8**	**p<0.01**	**53**	**10696**
OLANZAPINE	1.6	5.4	p = 0.02	30	7781
PALIPERIDONE	0.7	0.2	p = 0.653	3	1790
CLOZAPINE	0.6	1.6	p = 0.203	10	6234
**Hypnotic/Sedative**	3.9	68.5	p<0.01	97	10279
** TRIAZOLAM**	**8.7**	**40.3**	**p<0.01**	**7**	**325**
** ZOLPIDEM**	**6.7**	**222.1**	**p<0.01**	**48**	**2947**
** ESZOPICLONE**	**4.9**	**8.8**	**p<0.01**	**4**	**331**
** DIAZEPAM**	**3.1**	**13.7**	**p<0.01**	**11**	**1427**
** ALPRAZOLAM**	**3.0**	**17.5**	**p<0.01**	**15**	**2049**
** CLONAZEPAM**	**2.8**	**9.5**	**p<0.01**	**10**	**1462**
MIDAZOLAM	1.4	0	NA	1	292
LORAZEPAM	0.3	1.2	p = 0.269	1	1446

*Drugs associated with violence reports in **Table 1** **are in bold face.**

## Discussion

These data show that serious acts of violence towards others were regularly reported as an adverse drug event, and that marked differences were observed among drugs. Varenicline had the strongest association with violence by every measure used in this study. In addition, antidepressant drugs showed consistently elevated risk, even when compared with antipsychotics and mood stabilizers, which are used in psychiatric patients populations in which violent acts may occur. Violence cases as defined here were infrequently reported, accounting for 0.25% of all serious adverse drug events, and confined to a relatively small number of drugs.

This analysis shares many limitations common to studies based on spontaneously reported adverse drug events. The submission of an individual adverse event report does not itself establish causality, only that a reporting individual suspected a relationship existed. However, such reports frequently contribute to a broader assessment of causality. In the computer excerpts, the narrative description of each event is replaced by a series of standardized medical terms, as are adverse events in clinical studies. The quality and detail in each report varies, and the reporting rate for adverse drug events is unknown and believed to vary among types of event, among drugs and over periods of time. [15]

This study, however, contains numerous features intended to minimize the limitations of adverse event data from postmarketing surveillance. The proportional reporting ratio takes into account two possibilities: a) that wider use or a higher reporting rate exposes a drug to a greater chance of having a violence case attributed, and b) that a higher number of reports might have occurred by chance. The varying results among drugs for smoking cessation and the mood stabilizers show it is unlikely that the violence events are attributed to existing problems in the patient populations treated. Also, the focus of this study was on specific event terms that unequivocally described a violent act or thought – such as homicide or physical assault. By excluding more general adverse event terms such as “aggression” or “anger” many thousands of less specific cases were eliminated under the study criteria. While this means that the study did not count many possible cases of violence towards others (a loss of sensitivity) the restrictive criteria increased specificity. However, given that violent thoughts or actions are not typically attributed to drug therapy or recorded in medical records, the reporting rate for violence cases could be very low. The selected violence cases do not provide a reliable estimate of how often they might occur.

### Common and Unusual Features

These events were reported in a patient population that was 41% female and one-half older than age 36. In addition a majority of reports were submitted either by health professionals (primarily MDs) or from foreign sources where reporting is normally limited to health professionals.

While the reported events occurred among drugs used in widely different patient populations, the list of suspects was dominated by drugs that increase the availability of serotonin or dopamine in the brain. Most of the antidepressants increase the availability of serotonin through reuptake inhibition. Varenicline increases the availability of dopamine through partial antagonism of acetylcholine nicotinic receptors. [16] Sodium oxybate is a dopamine agonist indicated for narcolepsy. [17] Amphetamines increase concentrations of dopamine and serotonin. [18] On the other hand, no signal was seen for many common mood stabilizers such valproic acid, carbamazepine, and phenytoin, even though these drugs are used in bipolar patients who may experience psychosis in the acute manic phase and therefore be more prone to violence.

The proportional reporting ratio—our primary measure of elevated risk—showed consistency among drugs with the most similar mechanisms of action. As shown in Table 3, for example, venlafaxine had a PRR of 8.3 and desvenlafaxine a PRR of 7.9 even though the number of total cases and violence cases were different. Similarly citalopram had a PRR of 4.3 and escitalopram a PRR of 5.0. We believe it is also noteworthy that no signal whatever was seen for an overwhelming majority of drugs.

### Additional Questions for Study

We have previously examined varenicline's association with serious psychiatric symptoms including aggression/violence. [19]–[22] The aggression/violence case series for varenicline was consistent with these data but revealed other features that may or may not occur in cases attributed to other drugs. These features include early onset of psychiatric symptoms (usually within a few days), a senseless act of aggression/violence directed at anyone who happened to be near by, and resolution of the symptoms upon discontinuation. A more detailed case series of several hundred cases involving different classes of drugs would greatly improve scientific understanding of this drug adverse event and possibly lead the way towards identifying an at-risk patient population.

### Conclusions

These data provide new evidence that acts of violence towards others are a genuine and serious adverse drug event that is associated with a relatively small group of drugs. Varenicline, which increases the availability of dopamine, and serotonin reuptake inhibitors were the most strongly and consistently implicated drugs. Prospective studies to evaluate systematically this side effect are needed to establish the incidence, confirm differences among drugs and identify additional common features.

## References

[1] Mosholder AD, Pamer CA (2006). Postmarketing surveillance of suicidal adverse events with pediatric use of antidepressants.. J Child Adolesc Psychopharmacol.

[2] Mosholder AD, Willy M (2006). Suicidal adverse events in pediatric randomized, controlled clinical trials of antidepressant drugs are associated with active drug treatment: a meta-analysis.. J Child Adolesc Psychopharmacol.

[3] Pfizer Inc (2010). CHANTIX (varenicline tartrate) tablet, film coated [package insert]..

[4] Sanofi-Adventis US (2009). AMBIEN (zolpidem tartrate) tablet, film coated [package insert]..

[5] Merck & Co. I (2010). SINGULAIR (montelukast sodium) tablet, film coated [package insert]..

[6] U.S.Food and Drug Administration (2007). New Warnings Proposed for Antidepressants.. http://www.fda.gov/ForConsumers/ConsumerUpdates/ucm048950.htm.

[7] GlaxoSmithKline (2009). PAXIL (paroxetine hydrochloride) tablet, film coated [package insert]..

[8] Pfizer Inc (2008). ZOLOFT (sertraline hydrochloride) tablet, film coated [package insert]..

[9] AstraZeneca Pharmaceuticals LP (2010). SEROQUEL (quetiapine fumarate) tablet, film coated [package insert]..

[10] Moore T, Cohen M, Furberg C (2008). QuarterWatch: 2008 Quarter 1.. http://www.ismp.org/QuarterWatch/2008Q1.pdf.

[11] U.S.Food and Drug Administraton (2010). The Adverse Event Reporting System (AERS): Latest Quarterly Data File.. http://www.fda.gov/Drugs/GuidanceComplianceRegulatoryInformation/Surveillance/ucm082193.

[12] National Library of Medicine (2010). Unified Medical Language System (UMLS): RxNorm.. http://www.nlm.nih.gov/research/umls/rxnorm/.

[13] MedDRA Maintenance and Support Organization (2009). Introductory Guide: MedDRA Version 12.1..

[14] Evans SJ, Waller PC, Davis S (2001). Use of proportional reporting ratios (PRRs) for signal generation from spontaneous adverse drug reaction reports.. Pharmacoepidemiol Drug Saf.

[15] Moore TJ, Cohen MR, Furberg CD (2010). QuarterWatch: 2009 Quarter 4.. http://www.ismp.org/QuarterWatch/2009Q4.pdf.

[16] Coe JW, Brooks PR, Vetelino MG, Wirtz MC, Arnold EP (2005). Varenicline: an alpha4beta2 nicotinic receptor partial agonist for smoking cessation.. J Med Chem.

[17] Jazz Pharmaceuticals (2009). XYREM (sodium oxybate) solution [package insert]..

[18] Goodman LS, Hardman JG, Limbird LE, Gilman AG (2001). Goodman & Gilman's the Pharmacological Basis of Therapeutics..

[19] (2008). Varenicline: a British review. Unfavorable risk-benefit balance.. Prescrire Int.

[20] Moore TJ, Furberg CD (2009). Varenicline and suicide. Risk of psychiatric side effects with varenicline.. BMJ.

[21] Moore TJ, Glenmullen J, Furberg CD (2010). Thoughts and acts of aggression/violence toward others reported in association with varenicline.. Ann Pharmacother.

[22] Moore TJ, Cohen MR, Furberg CD (2008). Strong Signal Seen for New Varenicline Risks.. http://ismp.org/QuarterWatch/chantixReport.asp.

